# Effect of cervical spine motion on displacement of posterolateral annulus fibrosus in cervical spondylotic radiculopathy with contained posterolateral disc herniation: a three-dimensional finite element analysis

**DOI:** 10.1186/s13018-022-03450-5

**Published:** 2022-12-18

**Authors:** Lin-qiang Ye, Chao Chen, Yuan-hui Liu, Zhen Li, Guo-liang Lu

**Affiliations:** Department of Orthopaedics, Dongguan Hospital of Traditional Chinese Medicine, Dongguan, Guangdong China

**Keywords:** Cervical spondylotic radiculopathy, Cervical spine motion, Three-dimensional finite element analysis, Displacement of posterolateral annulus fibrosus

## Abstract

**Background:**

Previous studies on dynamic impingement of nerve root in cervical spondylotic radiculopathy (CSR) have focused on effect of cervical spine motion (CSM) on dimensional changes of intervertebral foramen. However, there are few studies to investigate effect of CSM on displacement of posterolateral intervertebral disc until now. The present study aimed to investigate effect of CSM on displacement of posterolateral annulus fibrosus (AF) in CSR with contained posterolateral disc herniation.

**Methods:**

A C5–C6 CSR finite element model with unilateral contained posterolateral disc herniation was generated based on validated C5–C6 normal finite element model. Forward and backward displacement distributions of posterolateral AFs in CSR model and normal model were compared. Changes in forward and backward displacement magnitudes of posterolateral AFs of the herniated side and the healthy side in CSR model, with respect to those of the ipsilateral posterolateral AFs in normal model, were compared. The comparisons were performed under flexion, extension, lateral bendings and axial rotations.

**Results:**

There was no difference in deformation trend of posterolateral AF between CSR model and normal model. Bilateral posterolateral AFs mainly moved forward during flexion and backward during extension. Left posterolateral AF mainly moved backward and right posterolateral AF forward during left lateral bending and left axial rotation. Left posterolateral AF mainly moved forward and right posterolateral AF backward during right lateral bending and right axial rotation. However, with respect to forward and backward displacement magnitudes of the ipsilateral posterolateral AFs in normal model, those of the herniated side increased relatively significantly compared with those of the healthy side in CSR model.

**Conclusions:**

Flexion, lateral bending to the healthy side and axial rotation to the healthy side make posterolateral AF of the herniated side mainly move forward, whereas extension, lateral bending to the herniated side and axial rotation to the herniated side make it mainly move backward. These data may help select CSM or positions to diagnose and treat CSR with contained posterolateral disc herniation. Increase in deformation amplitude of posterolateral AF of the herniated side may also be the reason for dynamic impingement of nerve root in CSR with contained posterolateral disc herniation.

## Background

Cervical spondylotic radiculopathy (CSR) is the most common cervical degenerative disease with an incidence of 107.3 per 100,000 for men and 63.5 per 100,000 for women [1, 2]. Although the exact pathophysiology of CSR is not completely understood, mechanical compression of nerve root combined with inflammatory changes due to cervical foraminal stenosis is often thought of the key factor leading to pain and neurologic dysfunction [3, 4]. Cervical foraminal stenosis is commonly caused by cervical posterolateral disc herniation and cervical spondylosis [5].

It is believed that in addition to static factors, dynamic factors in the cervical spinal column affect the amount of nerve root compression. Clinicians commonly employ cervical spine motion (CSM) for diagnostic evaluation and subsequent treatment methods of CSR [6–8]. Proper CSM or positions most likely will facilitate pain relief. Previous studies on the effects of CSM on diagnosis and treatment of CSR have focused on effect of CSM on dimensional changes of intervertebral foramen [8–11]. Flexion of cervical spine lengthens intervertebral foramen, whereas extension shortens intervertebral foramen. Rotation to the ipsilateral side narrows intervertebral foramen, whereas rotation to the contralateral side widens intervertebral foramen.

Since posterolateral disc herniation is one of the important pathological factors of CSR and may exist alone in some CSR patients [3], displacement of posterolateral intervertebral disc caused by CSM may also lead to dynamic impingement of the nerve root in CSR. However, there are few studies to investigate effect of CSM on displacement of posterolateral intervertebral disc until now. Relative to displacement of nucleus pulposus, finite element (FE) analysis can accurately reflect the displacement of annulus fibrosus (AF) [12]. In addition, the bulging posterolateral AF is the main structure of nerve root compression in contained posterolateral disc herniation. Understanding the influence of CSM on displacement of posterolateral AF may also provide a reference for clinicians to use CSM or positions in the diagnosis and treatment of CSR with contained posterolateral disc herniation from the perspective of displacement of AF. Therefore, the purpose of this study was to investigate effect of CSM on displacement of posterolateral AF in CSR with contained posterolateral disc herniation through three-dimensional FE analysis.

## Methods

### Construction of C5–C6 normal FE model

**Fig. 1:**
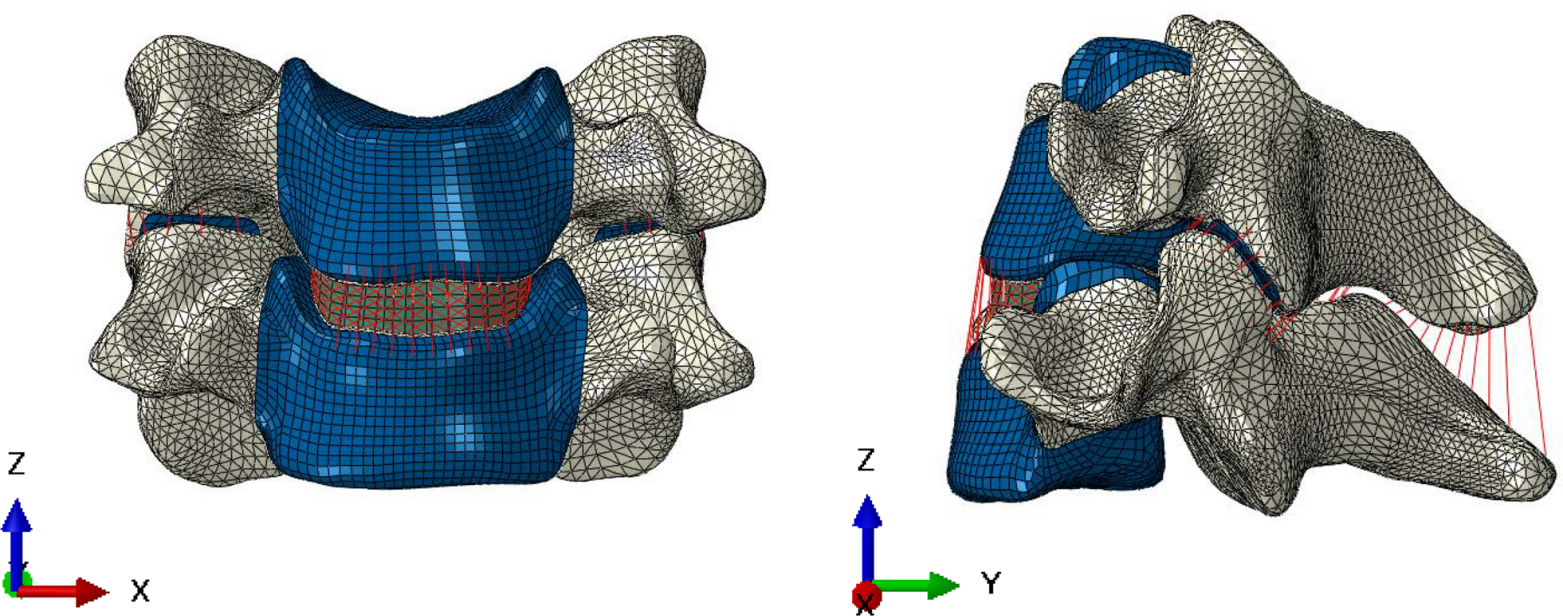
The C5–C6 normal FE model

A normal three-dimensional digital anatomical FE model of C5–C6 was created based on CT images of a C5–C6 motion segment without injury or radiographic evidence of degeneration. The image data were from a healthy male volunteer (25 years old, 65 kg and 172 cm). The study plan was approved by the ethics committee of our hospital. The participant was explained about the research purpose and signed the consent form. Slice thickness was 0.625 mm. The slice images were preserved in a computer and then imported to Mimics 19.0 software (Materialise Inc., Belgium) for generation of the three-dimensional geometries of C5–C6 vertebra. Then, a smoothing process was performed in Geomagic Studio 2013 software (Geomagic Inc., USA) to remove spikes and holes on the surface of the vertebral geometries. The geometries of other structures including AF, nucleus pulpous, facet cartilages and endplates, which were difficult to be separated from the CT images, were created using SolidWorks 2017 sofeware (SolidWorks Corp. USA). Each vertebra consisted of a cancellous core surrounded by a cortical shell layer with thickness of 0.4 mm [13]. Cartilaginous endplates were simulated with thickness of 0.2 mm at both ends of each vertebra [14]. The nucleus pulpous occupied about 25% of the total disc [15]. Facet joints were modeled with a cartilage layer with thickness of 0.5 mm and a gap of about 0.25 mm [16]. In order to mimic collagen fibers of AF, five layers of collagen fibers were embedded radially in the annular ground substances. For each layer, two bundles of fibers were modeled with orientation of about ± 25° with respect to the horizontal plane using truss elements [14]. Element types of cancellous bone, posterior bony element, cartilaginous endplates, AF and nucleus pulpous were defined as solid elements. Cortical bone and facet joint cartilage were defined as shell elements. Six different ligaments including anterior longitudinal ligament, posterior longitudinal ligament, interspinous ligament, supraspinal ligament, capsular ligament, and ligamentum flavum in tension only were modeled with truss elements. The assigned material properties were assumed to be homogeneous, linear, and isotropic. Truss elements were tension-only. Tied constraints were used to ensure disc and ligament attachments to the vertebra and prevent any relative movement during the simulations. Surface-to-surface contact algorithm was used in defining facet joint interaction, and friction coefficient was assumed to be 0.1 [15]. Material properties were taken from the studies and are listed in Table 1 [14, 15, 17]. The total number of nodes and elements was 283,423 and 188,728, respectively (Fig. 1). Convergence within 1% was achieved in the normal model to make sure that the results based on the present mesh density approached a reasonable solution [13].

**Table 1:** Material properties of C5–C6 finite element models

Component name	Young’s modulus (MPa)	Poisson’s ratio	Cross-sectional area (mm^2^)
Cortical bone	12,000	0.29	–
Cancellous bone	450	0.29	–
Posterior element	3500	0.29	–
Cartilage	10.4	0.4	–
Endplate	500	0.4	–
Nucleus pulposus	1	0.49	–
Annulus ground substance	3.4	0.4	–
Annulus fiber	110	0.3	1
ALL	10	0.3	6.0
PLL	10	0.3	5.0
CL	10	0.3	46.0
FL	1.5	0.3	5.0
ISL	1.5	0.3	10
SSL	1.5	0.3	5.0

ALL, anterior Longitudinal ligament; PLL, posterior longitudinal ligament; CL, capsular ligament; FL, flaval ligament; ISL, interspinous ligament; SSL, supraspinous ligament

### Construction of C5–C6 CSR FE model with unilateral contained posterolateral disc herniation

**Fig. 2:**
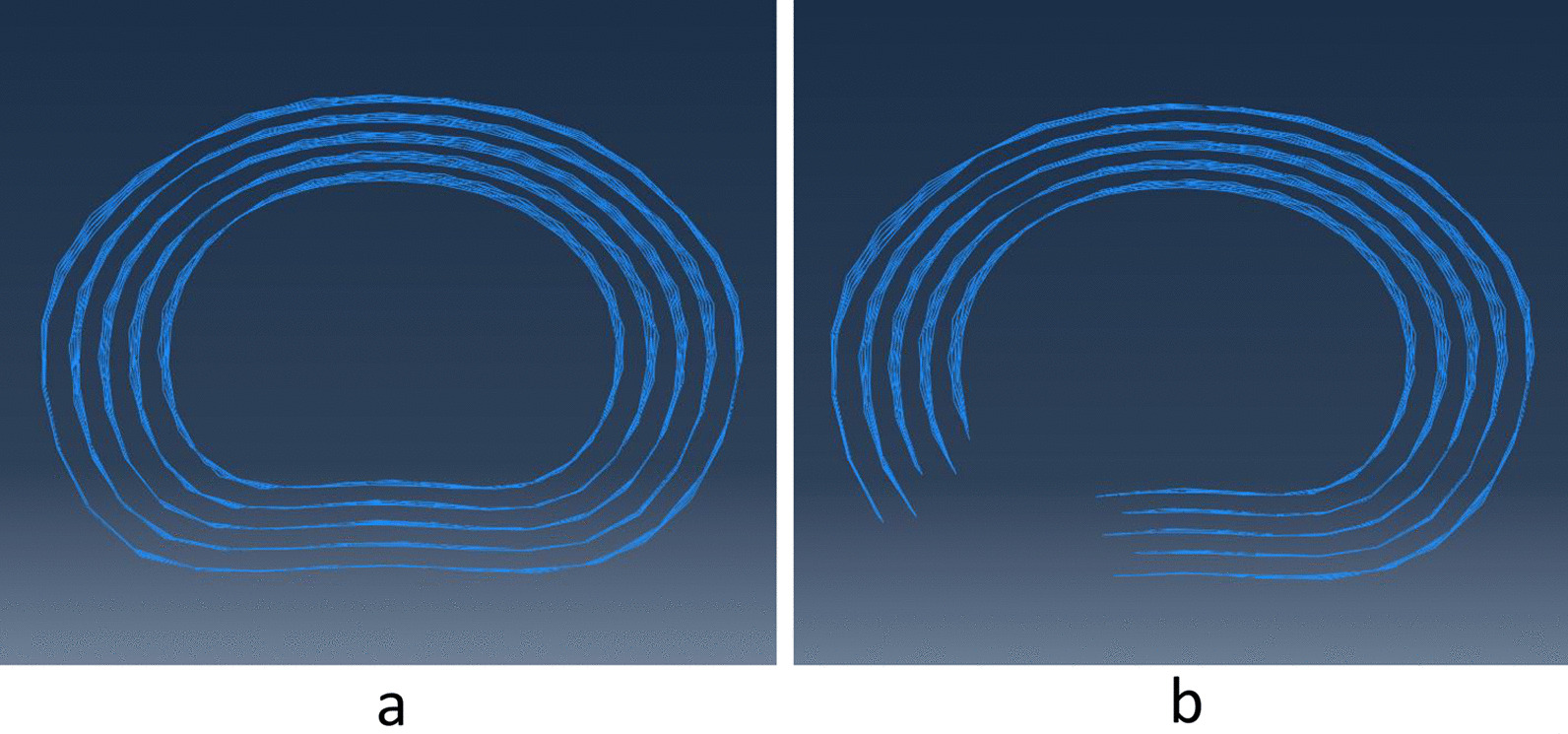
Models of intact annular fiber (**a**) and left posterolateral annular fiber defect (**b**)

To investigate effect of CSM on displacement of posterolateral AF in CSR with contained posterolateral disc herniation, unilateral contained posterolateral disc herniation was simulated based on the developed C5–C6 normal FE model. Partial damage of left posterolateral AF with limited nuclear migration in the earliest phase of disc herniation was modeled by removing annular fibers of left posterolateral AF and preserving the annulus ground substance without constructing geometrical morphology of posterolateral disc herniation (Fig. 2).

### Boundary and loading conditions of the normal and CSR FE models

The inferior endplate of C6 was fixed at six degrees of freedom. A axial pre-compression load of 73.6 N was applied to the superior endplate center of C5 for head physical gravity simulation, and moment of 1.8 Nm on sagittal, coronal and transverse anatomical planes to simulate the primary spinal motions of flexion, extension, lateral bendings and axial rotations [16, 18]. All computational processes were performed with Abaqus 2016 software (Simulia Inc., USA).

### Analysis

Forward and backward (Y direction) displacement distributions of posterolateral AFs in CSR model and normal model were compared. The same nodes were selected as reference points for forward and backward displacement magnitudes of bilateral posterolateral AFs in CSR model and normal model. Changes in forward and backward displacement magnitudes of posterolateral AFs of the herniated side and the healthy side in CSR model, with respect to those of the ipsilateral posterolateral AFs in normal model, were compared. The comparisons were performed under flexion, extension, lateral bendings and axial rotations.

## Results

### Validation of the normal FE model

**Fig. 3:**
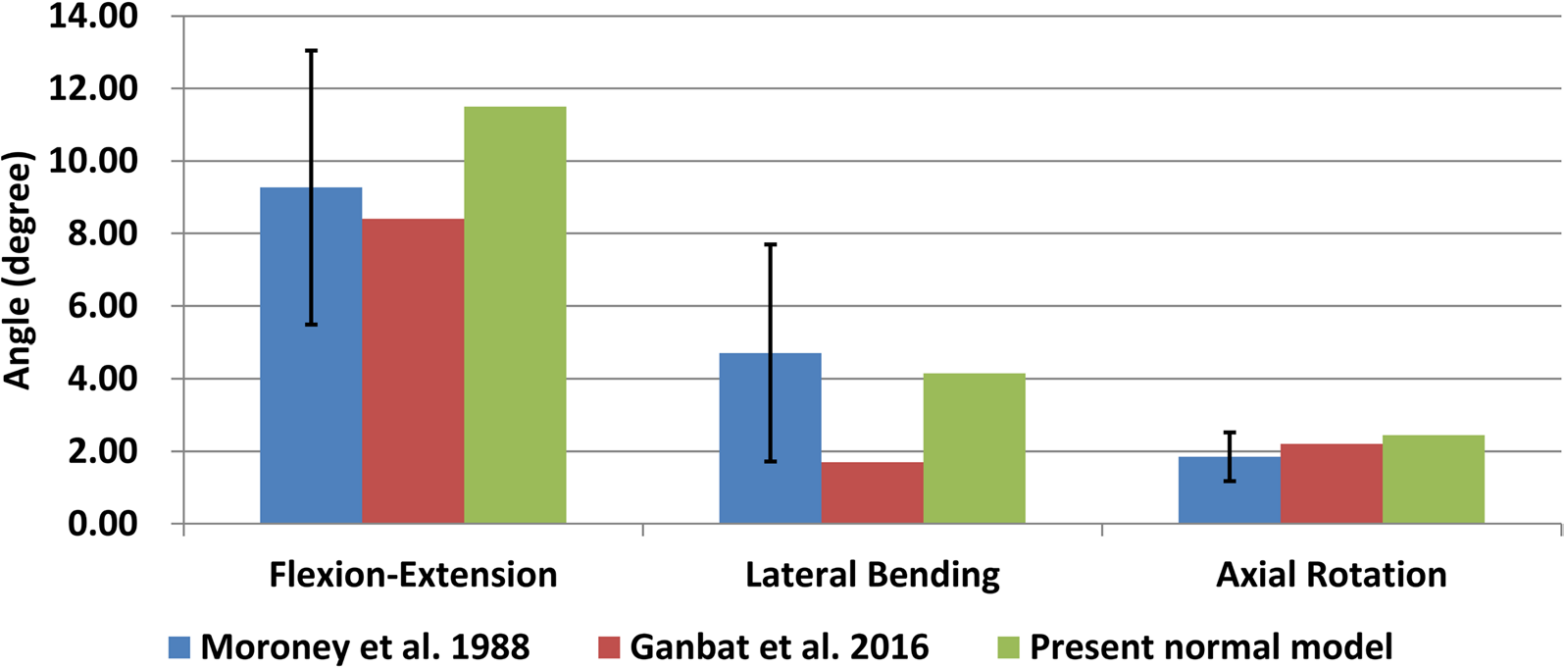
Validation of C5–C6 normal FE model

Under 73.6 N follower load and 1.8 Nm moment, the ROMs of C5–C6 were 7.0° in flexion, 4.5° in extension, 3.6° in left lateral bending, 4.7° in right lateral bending, 2.1° in left axial rotation and 2.8° in right axial rotation, which were similar to those published in studies (Fig. 3) [16, 18].

### Forward and backward displacement distributions of posterolateral AFs

**Fig. 4:**
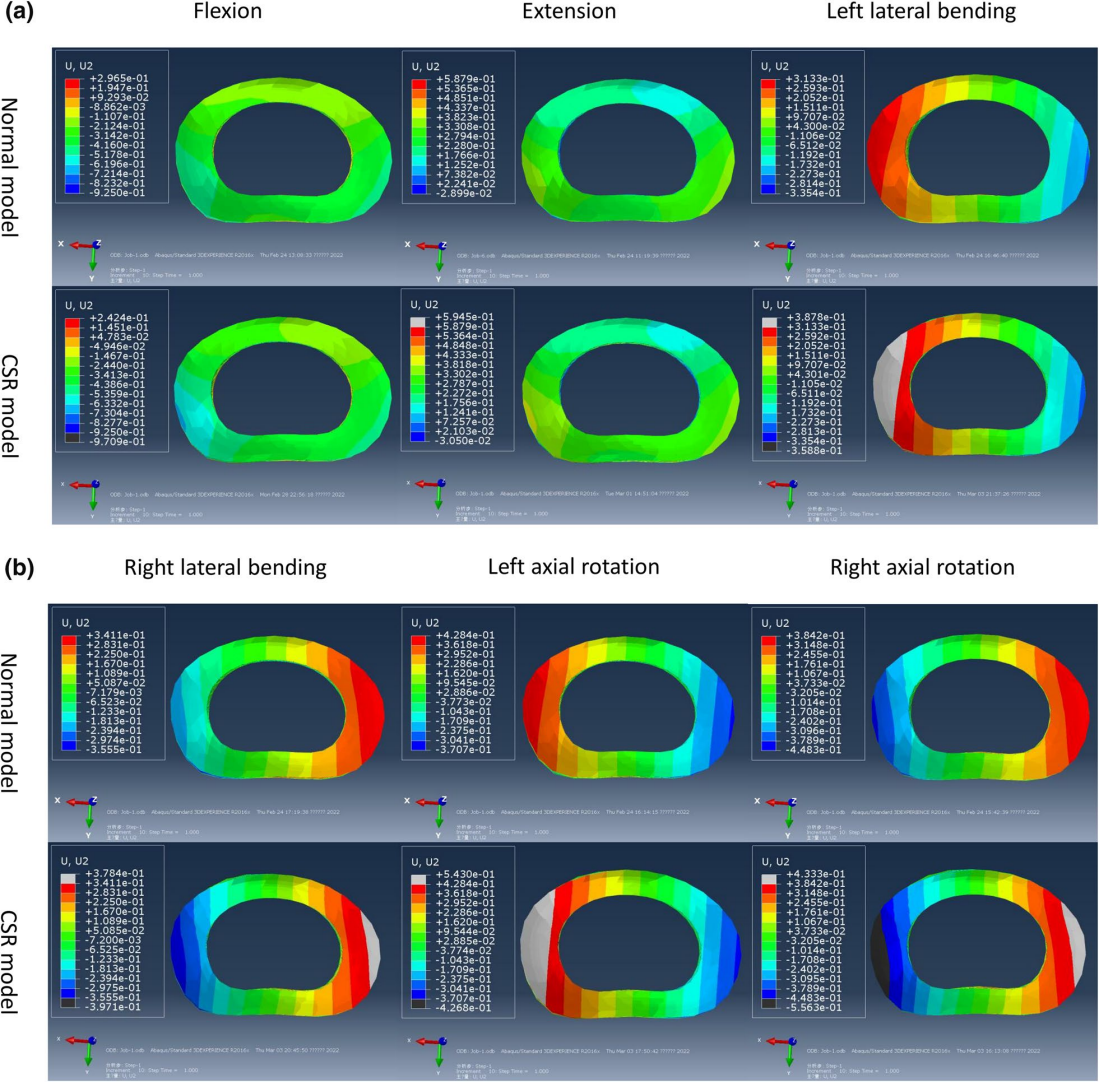
Nephograms of forward and backward displacement distributions of AFs in normal model and CSR model under flexion, extension, left lateral bending (**a**) and right lateral bending, left axial rotation, right axial rotation (**b**)

There was no difference in deformation trend of posterolateral AF between CSR model and normal model under all loading conditions. Bilateral posterolateral AFs mainly moved forward during flexion and backward during extension. Left posterolateral AF mainly moved backward and right posterolateral AF forward during left lateral bending and left axial rotation. Left posterolateral AF mainly moved forward and right posterolateral AF backward during right lateral bending and right axial rotation (Fig. 4).

### Forward and backward displacement magnitudes of posterolateral AFs

**Fig. 5:**
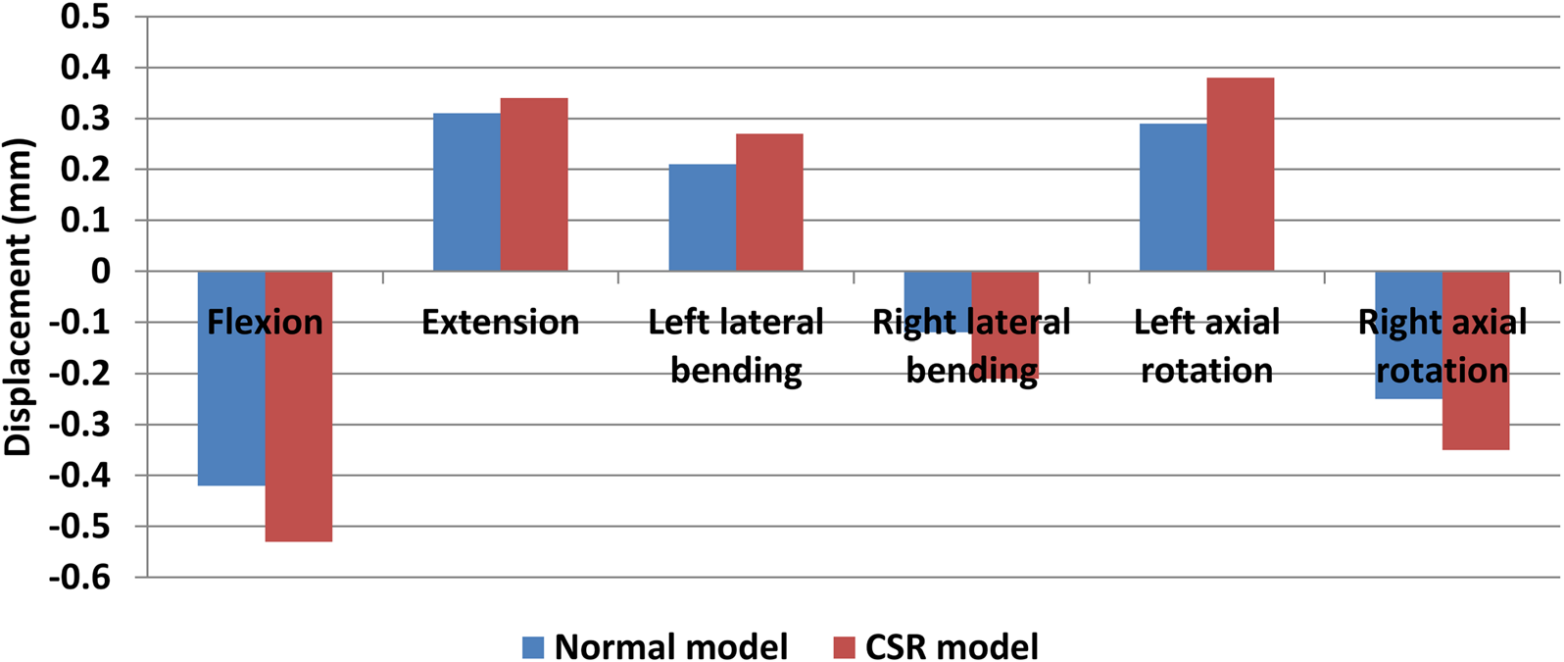
Forward (negative) and backward (positive) displacements of posterolateral AF of the herniated side in CSR model and those of the ipsilateral side in normal model

**Fig. 6:**
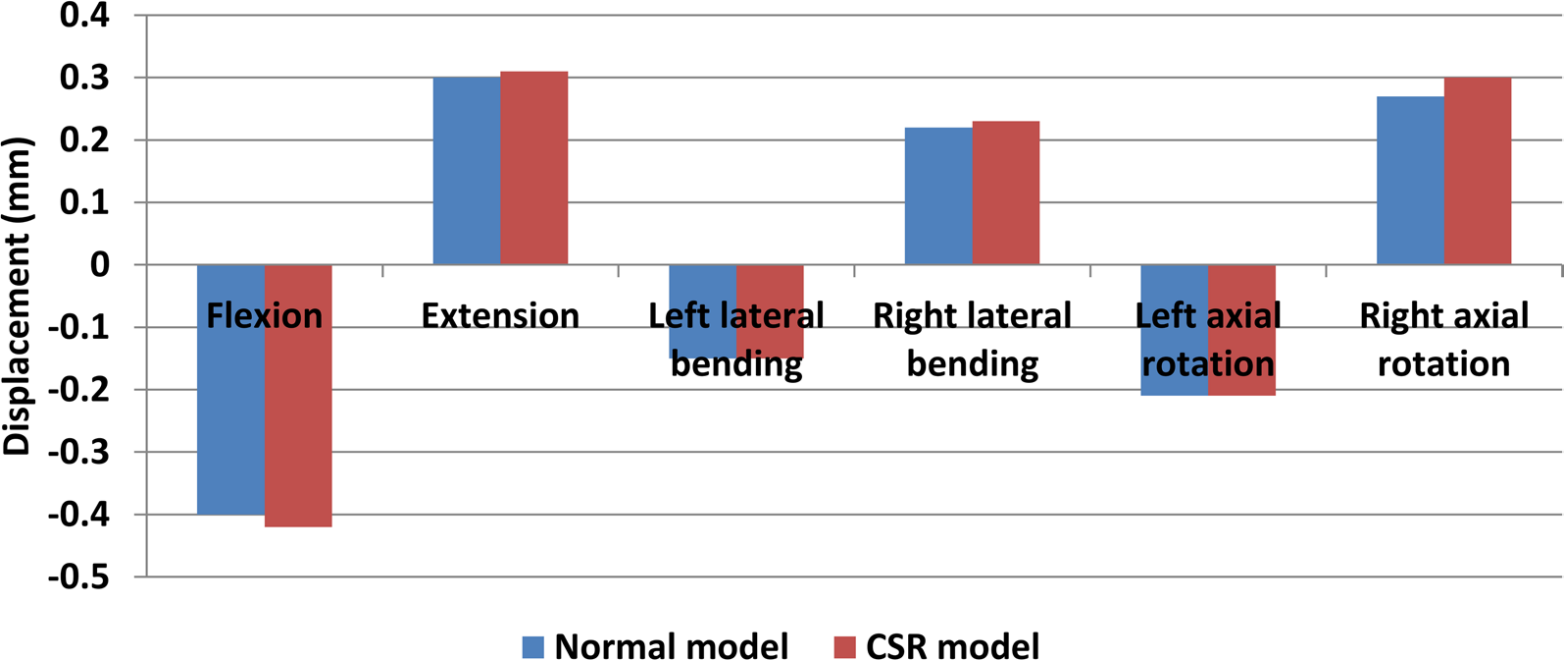
Forward (negative) and backward (positive) displacements of posterolateral AF of the healthy side in CSR model and those of the ipsilateral side in normal model

Forward and backward displacement magnitudes of left posterolateral AF were − 0.42 mm, 0.31 mm, 0.21 mm, − 0.12 mm, 0.29 mm and − 0.25 mm while those of right posterolateral AF were − 0.4 mm, 0.3 mm, − 0.15 mm, 0.22 mm, − 0.21 mm and 0.27 mm under flexion, extension, left lateral bending, right lateral bending, left axial rotation and right axial rotation, respectively, in normal model. Forward and backward displacement magnitudes of left posterolateral AF were − 0.53 mm, 0.34 mm, 0.27 mm, − 0.21 mm, 0.38 mm and − 0.35 mm while those of right posterolateral AF were − 0.42 mm, 0.31 mm, − 0.15 mm, 0.23 mm, − 0.21 mm and 0.3 mm under flexion, extension, left lateral bending, right lateral bending, left axial rotation and right axial rotation, respectively, in CSR model. With respect to forward and backward displacement magnitudes of the ipsilateral posterolateral AFs in normal model, those of the herniated side increased relatively significantly (Fig. 5) compared with those of the healthy side in CSR model (Fig. 6).

## Discussion

The influence of CSM or position on displacement of posterolateral AF has already been employed in some treatment methods of CSR with contained posterolateral disc herniation such as manipulation [19], distraction [20] and surgical methods [21–23]. Huang et al. [19] investigated the biomechanical effects of cervical spinal manipulation on cervical disc herniation using three-dimensional FE analysis and found that cervical spinal manipulation with rotation to the healthy side made posterolateral AF of the herniated side move forward, which may help to change the relative position of posterolateral AF and nerve root to release adhesion and relieve compression [24]. Wong et al. [20] studied the influence of different traction angles on intervertebral separation and found that posterior intervertebral space in flexion position before traction was greater than that in the neutral position, which indicates that flexion position had a pulling effect on the posterolateral AF and may contribute to make bulging posterolateral AF move forward. Geol et al. [21, 22] treated CSR with interfacet distraction and fusion and postoperative follow-up MRI showed disappearance of disc herniation. It is probable that interfacet distraction caused longitudinal intervertebral separation and mild flexion of superior vertebrae, which had a pulling effect on the posterolateral AF and make bulging posterolateral AF move forward [21–23]. Thus, understanding the influence of CSM on displacement of posterolateral AF can help clinicians choose proper CSM or positions for some treatment methods of CSR with contained posterolateral disc herniation from the perspective of displacement of AF.

Although the research object of our study was CSR with contained posterolateral disc herniation, various degrees of protrusion of nucleus pulposus toward AF do exist. However, our study focused on variation tendency rather than precise numerical values of displacement of posterolateral AF caused by CSM in CSR with contained posterolateral disc herniation, which belongs to a qualitative study rather than a quantitative study. In addition, microstructural analysis of disc herniation found that microstructural changes of disc herniation in the earliest phase were partial damage of AF and limited nuclear migration [25]. Therefore, we believed that the key point of pathologic model construction for our study was simulating partial damage of posterolateral AF but not the geometrical morphology of posterolateral disc herniation. For these reasons, Partial damage of left posterolateral AF with limited nuclear migration in the earliest phase of disc herniation was modeled by removing annular fibers and preserving the annulus ground substance of left posterolateral AF without constructing geometrical morphology of posterolateral disc herniation. We acknowledge that the CSR model constructed in our study is a simplified model that cannot completely simulate all the pathological characteristics of CSR, but it can meet research requirements of our study. Forward and backward displacement of posterolateral AF has been proposed as a parameter of dynamic impingement of nerve root caused by bulging posterolateral AF in CSR with contained posterolateral disc herniation [19].

The validation test proved that the constructed C5–C6 normal FE model could accurately simulate physiological activities of C5–C6 functional unit [16, 18]. The results of our study also showed that displacement magnitudes of posterolateral AFs were from 0.12 to 0.42 mm in the normal model, which are consistent with numerical values of previous studies [12, 19], indicating that the normal model constructed in our study can accurately analyze displacement magnitude of posterolateral AF. Therefore, the normal model could be a valuable tool for later research.

The result of forward and backward displacement distributions of posterolateral AFs showed that there was no difference in deformation trend of posterolateral AF between CSR model and normal model under all loading conditions. Bilateral posterolateral AFs mainly moved forward during flexion and backward during extension. Left posterolateral AF mainly moved backward and right AF forward during both left lateral bending and left axial rotation. Left AF mainly moved forward and right AF backward during both right lateral bending and right axial rotation. This result indicates that flexion, lateral bending to the healthy side and axial rotation to the healthy side make posterolateral AF of the herniated side mainly move forward, whereas extension, lateral bending to the herniated side and axial rotation to the herniated side make it mainly move backward. Therefore, flexion, lateral bending to the healthy side and axial rotation to the healthy side may relieve compression of nerve root by posterolateral AF of the herniated side, whereas extension, lateral bending to the herniated side and axial rotation to the herniated side may aggravate it in CSR with contained posterolateral disc herniation. The result of forward and backward displacement magnitudes of posterolateral AFs showed that with respect to forwnd backward displacement magnitudes of the ipsilateral posterolateral AFs in normal model, those of the ard aherniated side increased relatively significantly compared with those of the healthy side in CSR model. This result indicates that deformation amplitude of posterolateral AF of the herniated side is larger than that of the healthy side. Therefore, increase in deformation amplitude of posterolateral AF of the herniated side may also be the reason for dynamic impingement of nerve root in CSR with contained posterolateral disc herniation.

Some limitations of this study need to be mentioned. Firstly, the assumptions of linear, isotropic, homogeneous material properties for FE models were a simplification of the real situation. Secondly, effect of CSM on displacement of nucleus pulposus is not involved in this study because FE analysis can not accurately reflect the displacement of nucleus pulposus. Hence, conclusion of this study is only suitable for CSR with contained posterolateral disc herniation. Thirdly, this study only focused on the effect of simple CSM such as flexion, extension, lateral flexion and axial rotation on displacement of AF in CSR. Future investigation concerning complex CSM on displacement of AF is needed if necessary. Fourthly, the muscular influence on displacement of AF is not considered in this study. It remains unknown how much the current findings would vary if this limitation is appropriately addressed.

## Conclusions

Flexion, lateral bending to the healthy side and axial rotation to the healthy side make posterolateral AF of the herniated side mainly move forward, whereas extension, lateral bending to the herniated side and axial rotation to the herniated side make it mainly move backward. These data may have implications for selection of CSM or positions to diagnose and treat CSR with contained posterolateral disc herniation. Increase in deformation amplitude of posterolateral AF of the herniated side may also be the reason for dynamic impingement of nerve root in CSR with contained posterolateral disc herniation.

## Data Availability

The datasets used and/or analyzed during the current study are available from the corresponding author or the first author on reasonable request.

## Abbreviations

CSR: Cervical spondylotic radiculopathy
CSM: Cervical spine motion
FE: Finite element
ROM: Rang of motion
CT: Computed tomography
AF: Annulus fibrosus

## References

[1] Radhakrishnan K, Litchy WJ, O'Fallon WM (1994). Epidemiology of cervical radiculopathy. A population-based study from Rochester, Minnesota, 1976 through 1990. Brain.

[2] Woods BI, Hilibrand AS (2015). Cervical radiculopathy: epidemiology, etiology, diagnosis, and treatment. J Spinal Disord Tech.

[3] Abbed KM, Coumans JV (2007). Cervical radiculopathy: pathophysiology, presentation, and clinical evaluation. Neurosurgery.

[4] Garfin SR, Rydevik B, Lind B (1995). Spinal nerve root compression. Spine (Phila Pa 1976).

[5] Hirai S, Kato S, Nakajima K (2021). Anatomical study of cervical intervertebral foramen in patients with cervical spondylotic radiculopathy. J Orthop Sci.

[6] Gudavalli S, Kruse RA (2008). Foraminal stenosis with radiculopathy from a cervical disc herniation in a 33-year-old man treated with flexion distraction decompression manipulation. J Manipulative Physiol Ther.

[7] Siemionow K, Janusz P, Glowka P (2016). Cervical cages placed bilaterally in the facet joints from a posterior approach significantly increase foraminal area. Eur Spine J.

[8] Yoo JU, Zou D, Edwards WT (1992). Effect of cervical spine motion on the neuroforaminal dimensions of human cervical spine. Spine (Phila Pa 1976).

[9] Kitagawa T, Fujiwara A, Kobayashi N (2004). Morphologic changes in the cervical neural foramen due to flexion and extension: in vivo imaging study. Spine (Phila Pa 1976).

[10] Mao H, Driscoll SJ, Li JS (2016). Dimensional changes of the neuroforamina in subaxial cervical spine during in vivo dynamic flexion-extension. Spine J.

[11] Muhle C, Resnick D, Ahn JM (2001). In vivo changes in the neuroforaminal size at flexion-extension and axial rotation of the cervical spine in healthy persons examined using kinematic magnetic resonance imaging. Spine (Phila Pa 1976).

[12] Krag MH, Seroussi RE, Wilder DG (1987). Internal displacement distribution from in vitro loading of human thoracic and lumbar spinal motion segments: experimental results and theoretical predictions. Spine (Phila Pa 1976).

[13] Mo ZJ, Zhao YB, Wang LZ (2014). Biomechanical effects of cervical arthroplasty with U-shaped disc implant on segmental range of motion and loading of surrounding soft tissue. Eur Spine J.

[14] Lee SH, Im YJ, Kim KT (2011). Comparison of cervical spine biomechanics after fixed- and mobile-core artificial disc replacement: a finite element analysis. Spine (Phila Pa 1976).

[15] Wang Z, Zhao H, Liu JM (2016). Resection or degeneration of uncovertebral joints altered the segmental kinematics and load-sharing pattern of subaxial cervical spine: a biomechanical investigation using a C2–T1 finite element model. J Biomech.

[16] Ganbat D, Kim YH, Kim K (2016). Effect of mechanical loading on heterotopic ossification in cervical total disc replacement: a three-dimensional finite element analysis. Biomech Model Mechanobiol.

[17] Ottardi C, Galbusera F, Luca A (2016). Finite element analysis of the lumbar destabilization following pedicle subtraction osteotomy. Med Eng Phys.

[18] Moroney SP, Schultz AB, Miller JA (1988). Load-displacement properties of lower cervical spine motion segments. J Biomech.

[19] Huang X, Ye L, Wu Z (2018). Biomechanical effects of lateral bending position on performing cervical spinal manipulation for cervical disc herniation: a three-dimensional finite element analysis. Evid Based Complement Altern Med.

[20] Wong AM, Leong CP, Chen CM (1992). The traction angle and cervical intervertebral separation. Spine (Phila Pa 1976).

[21] Goel A, Dharurkar P, Shah A (2019). Facetal fixation arthrodesis as treatment of cervical radiculopathy. World Neurosurg.

[22] Goel A, Shah A, Patni N (2016). Immediate postoperative reversal of disc herniation following facetal distraction-fixation surgery: report of 4 cases. World Neurosurg.

[23] Ramos MRD, Mendoza CJP, Yumol JV (2021). Multilevel, percutaneous posterior cervical interfacet distraction and fusion for cervical spondylotic radiculopathy: clinical and radiographic outcomes. Spine (Phila Pa 1976).

[24] Wu LP, Huang YQ, Manas D (2014). Real-time monitoring of stresses and displacements in cervical nuclei pulposi during cervical spine manipulation: a finite element model analysis. J Manipulative Physiol Ther.

[25] Wade KR, Robertson PA, Thambyah A (2014). How healthy discs herniate: a biomechanical and microstructural study investigating the combined effects of compression rate and flexion. Spine (Phila Pa 1976).

